# Hypothesis: increase of the ratio singlet oxygen plus superoxide radical to hydrogen peroxide changes stress defense response to programmed leaf death

**DOI:** 10.3389/fpls.2013.00479

**Published:** 2013-11-25

**Authors:** Bartolomé Sabater, Mercedes Martín

**Affiliations:** Departamento de Ciencias de la Vida (Fisiología Vegetal), Universidad de AlcaláMadrid, Spain

**Keywords:** chloroplast, chlororespiration, jasmonic acid, leaf senescence, programmed cell death, ROS, stress

## Abstract

The level of reactive oxygen species (ROS) increases under different stresses and, by destroying cellular components, may cause cell death. In addition, ROS are part of the complex network of transduction signals that induce defense reactions against stress or, alternatively, trigger programmed cell death, and key questions are the levels of each ROS that, respectively determine defense and death responses of the cell. The answer to those questions is difficult because there are several patterns of cell death that frequently appear mixed and are hardly distinguishable. Moreover, although considerable progresses have been achieved in the determination of the levels of specific ROS, critical questions remain on the ROS level in specific cell compartments. By considering chloroplasts as the main source of ROS in photosynthetic tissues at light, a comparison of the levels in stress and senescence of the chloroplastic activities involved in the generation and scavenging of ROS suggests plausible differences in the levels of specific ROS between stress defense and death. In effect, the three activities of the chlororespiratory chain increase similarly in stress defense response. However, in senescence, superoxide dismutase (SOD), that converts superoxide anion radical ($O_{2}^{∙-}$) to hydrogen peroxide (H_2_O_2_,) decreases, while the thylakoid Ndh complex, that favors the generation of singlet oxygen (^1^O_2_) and $O_{2}^{∙-}$, and peroxidase (PX), that consumes H_2_O_2_, increase. The obvious inference is that, in respect to defense response, the ratio (^1^O_2_ plus $O_{2}^{∙-}$)/H_2_O_2_ is increased in the senescence previous to cell death. We hypothesize that the different ROS ratios, probably through changes in the jasmonic acid/H_2_O_2_ ratio, could determine the activation of the defense network or the death network response of the cell.

## INTRODUCTION

Reactive oxygen species (ROS) were formerly known for their damage effects on cellular components. Later, evidences, mainly accumulated along the last decade of twentieth century, showed at the turn of the millennium (Potikha et al., 1999; Quirino et al., 2000; Van Breusegem et al., 2001; Apel and Hirt, 2004; Zimmermann and Zentgraf, 2005; Gechev et al., 2006; Zentgraf, 2007; Khanna-Chopra et al., 2013) additional roles of ROS as transduction signals within the complex network of molecules controlling developmental processes (mainly those leading to cell death) and responses to environmental stresses. In addition to the formidable problem to understand the functional integration of the complex networks regulating the different developmental processes with moderately stable node molecules as proteins and hormones, ROS signals posed entirely new challenges due to uncertainties on their generation and scavenging, on their mobility among cell compartments and on the molecular mechanisms of their interactions with other components (nodes) of the transduction network.

The involvement of ROS in the senescence of photosynthetic tissues provides a good system to size the magnitude of the challenges and to follow progresses to understand the integration of ROS signals within the networks controlling cell cycle and responses to stress. To distinguish between the ROS mediated effects in stress and in cell death, we address two fundamental aspects related to the roles of ROS in the programmed senescence of photosynthetic tissues under field conditions at light: (1) sources and sinks of ROS, and (2) signals that immediately follow ROS in the transduction networks. Programed senescence and death of photosynthetic leaves probably represents the highest amount of biomass and number of cells in the Earth suffering programmed cell death (PCD). Senescence of photosynthetic fruit tissues is a case of programmed senescence, not immediately followed of death, which is part of the maturation of most fleshy fruits and, as such, of high economic relevance. Similitude and differences between the senescence of leaf and the ripening of fruits were recognized since long time and we will refer the last exclusively for recent advances related to the involvement of components of the ROS generation machinery in fruit maturation. In a lesser extension, we will refer to a few processes where ROS signals are also involved and provide insights to understand leaf senescence under field conditions. Among them, the senescence and death of animal cell provides a lot of research advances that may be relevant. Senescence of non-photosynthetic plant cell, cell death associated to hypersensible response (HR), leaf senescence associated to diverse abiotic stresses (dark, drought, low, and high temperatures, nutrient deficiency,) and stress responses in general are fields where recent investigations provide insight relevant to the involvement of ROS as node signals in networks controlling cell processes.

There are different types of cell senescence and death, which frequently make difficult the comparison of the research results in different organisms, in different tissues of an organism and of different types of senescence and death of a specific tissue. For a comparison with the best characterized types of cell senescence and death in animals (Bialik et al., 2010), and in relation to the involvement of ROS, programmed leaf senescence and death shows similarities with the apoptotic/necrotic “intrinsic death pathways,” as far as the mitochondrial ROS source in animal is substituted by the chloroplast ROS source (Zapata et al., 2005; Doyle et al., 2010; Sabater and Martín, 2013).

## ROS IN PHOTOSYNTHESIZING LEAVES

**FIGURE 1:**
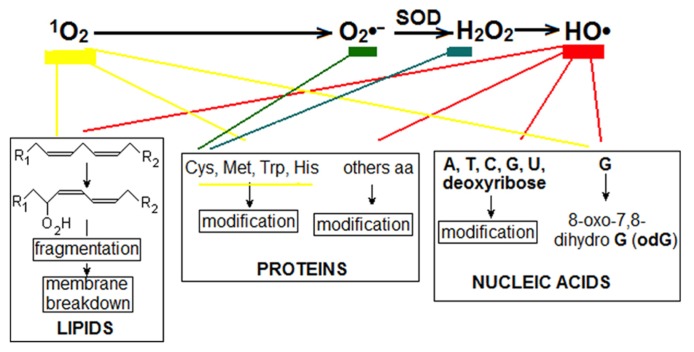
**Main ROS found in leaves.** Triplet excited chlorophyll (^3^Chl*) can transfer excitation to oxygen to produce singlet oxygen (^1^O_2_). Successive steps convert ^1^O_2_ to $O_{2}^{∙-}$, H_2_O_2_ and HO^∙^ . In addition, and probably mainly, $O_{2}^{∙-}$ is produced in the chloroplast by the transfer of one electron from reduced iron-sulfur proteins to O_2_ (Mehler reaction). Two $O_{2}^{∙-}$ and two H^+^, in reaction catalyzed by superoxide dismutase (SOD), produce one molecule of H_2_O_2_ and another of H_2_O. Decomposition of H_2_O_2_ to HO^∙^ is catalyzed by different divalent cations, especially Fe^2^^+^ (Fenton reaction). Alternatively, H_2_O_2_ may be consumed by catalase and peroxidase catalyzed reactions. Several cell components are destroyed by ROS. ^1^O_2_, directly, and HO^∙^ with oxygen transform 1,4-unsatured fatty acids to hydroperoxy-derivatives (-O_2_H) which further undergo different transformations, including fragmentations that disassemble membranes. Again, mainly ^1^O_2_ and HO^∙^ modify bases in DNA, RNA, and free bases, especially guanine (G) which is transformed to 8-oxo-7,8-dihydro guanine (odG), which can be paired with C or A, producing erroneous proteins and mRNA and DNA mutations. Most of the amino acids, free or in polypeptide chains, can be modified by ROS; cysteine is especially sensitive to $O_{2}^{∙-}$ and H_2_O_2_; cysteine, methionine, tryptophan, and histidine are especially sensitive to ^1^O_2_.

Sources and transformation of the main ROS has been intensely investigated in leaves and details may be consulted elsewhere (Karpinski et al., 2001; Sabater and Martín, 2013). **Figure 1** summarized the best-known ROS, their transformation and deleterious effects on cell components. In addition to the main ROS found in non-photosynthetic cells: superoxide anion radical ($O_{2}^{∙-}$), hydrogen peroxide (H_2_O_2_), and hydroxyl radical (HO^∙^); another ROS, the singlet oxygen (^1^O_2_), is produced in chloroplast by transfer of excitation from triplet excited chlorophyll (^3^Chl*) to O_2_. Hydroperoxyl radical (${HO}_{2}^{∙-}$) is also formed although probably in a lesser amount (Grace, 2005). The main four ROS are currently (and majorly) formed by chloroplasts at light and, to minimize their deleterious effects, scavenged by non-enzymatic and enzymatic reactions as superoxide dismutase (SOD), converting $O_{2}^{∙-}$ to H_2_O_2_, and peroxidase (PX) consuming H_2_O_2_.$O_{2}^{∙-}$, and then H_2_O_2_ and HO^∙^ , are also generated in other cell compartments, especially in mitochondria and peroxisomes where H_2_O_2_ is destroyed by catalase. To prevent the generation of excess ^1^O_2_, heat dissipation of ^3^Chl* excitation is enhanced through zeaxanthin formed by the xanthophyll cycle (Eskling et al., 2001). At high light intensity, the NADPH generated in the photosynthetic electron transport (PET) exceeds the capacity of the Benson–Calvin cycle to consume it (for example at low temperature or low CO_2_ supply by partially closed stomata). Then, transporters of the PET become over-reduced, the production of ROS increases and, depending on the environmental severity and the rapidity of the correction responses, it can destroy cell components producing the syndrome of photo-oxidative stress and eventually causing cell death (Levine, 1999).

It must be emphasized that over-reduction of components of PET causes increases of ROS whose destructive action is associated to their strong oxidant power and frequently is difficult to distinguish effects regulated by ROS from those regulated by the redox state of the chloroplast (Kruk and Szymánska, 2012). The paradox is not the unique problem to take into account when ROS are investigated as signal within the regulatory network of the cell. As pointed above, high level of ROS, *per se*, can produce cell death under stress conditions without involving specific signal transduction. To make more complex the situation, many evidences indicate that ROS signals are involved in the response to alleviate photo-oxidative stress that is mainly based on the repression of some genes and induction of some another, mainly those encoding the enzyme systems, as chloroplastic SODs, that scavenge ROS. Under this perspective, the key question is the level of each ROS required for such opposite cell responses as PCD and alleviation of stress to avoid death (Sabater and Martín, 2013). There is not yet a satisfactory response to that question, but there are some aspects, as the nature of the ROS acting as signal and the organelle location of sources and sinks of the different ROS, that provide valuable information on the signaling by ROS in leaf senescence.

## ARE ALL ROS TRANSDUCTION SIGNALS?

Damage effects of ROS (**Figure 1**) are mainly unspecific and could hardly be envisaged as related to their mechanism of interaction with other signal molecules of the regulatory network of the cell. However, effects other than damage are conceivable for H_2_O_2_. On the other hand, being ^1^O_2_, $O_{2}^{∙-}$ and HO^∙^ short-lived, the more stable (and permeable through membranes) H_2_O_2_ seems the plausible ROS signal candidate that interacts with other components of the regulatory cellular network and generates genetic and other responses of the cell to stress and PCD. Moreover, as H_2_O_2_ is substrate of catalase and diverse PXs, it could conceivably interact (enzymatic or not enzymatically) with proteins downstream in a signal cascade of a cellular network. H_2_O_2_ is also the product of the sequence of transformations of ^1^O_2_ and $O_{2}^{∙-}$ (**Figure 1**), which, in a first approximation, suggests that the increase of these ROS was finally senses by an increase of H_2_O_2_ and that, in the simplest imaginable mechanism, H_2_O_2_ would be the unique ROS signal of the cellular networks. From a broad perspective, the transformations ^1^O_2_ to $O_{2}^{∙-}$ and then to H_2_O_2_ should be no other than stages of signal transduction until the last ROS before non-ROS nodes. As we will see, facts are not so simple. Certainly, there many evidences that H_2_O_2_ is a signal, at least in the transduction cascades of the responses to stress, but there are strong evidences suggesting that $O_{2}^{∙-}$ and, specially, ^1^O_2_ could also be signals that, independently of their transformation to H_2_O_2_, originate cascade signals involved in leaf senescence.

## ROS GENERATION AND SCAVENGING IN STRESS AND SENESCENCE

In effect, the dynamic of ROS generation and scavenging in the healthy and mild-stressed leaves seems to change in senescence. As pointed above, in health photosynthesizing leaf, ^1^O_2_ and $O_{2}^{∙-}$ are formed in chloroplasts by excitation and electron transfer, respectively, to oxygen, processes that are enhanced when there is excess of light in respect to the capacity to consume NADPH (mainly in the Benson–Calvin cycle). Enzymatic and non-enzymatic mechanisms maintain low steady-state levels of ROS by lowering the formation of ^1^O_2_ through heath dissipation of excited chlorophyll and, in the reaction catalyzed by SOD, transforming $O_{2}^{∙-}$ to H_2_O_2_, which is consumed by PXs and transformed by the Fenton reaction (Fe^2^^+^ + H_2_O_2_→Fe^3^^+^ + HO^∙^ + OH^-^) to (Jakob and Heber, 1996). HO^∙^ is rapidly consumed in diverse reactions. In an unknown extension, ^1^O_2_ seems to be able to oxidize reduced plastoquinone contributing to draining electrons from over-reduced PET (Kruk and Szymánska, 2012). Among chloroplast PXs, plastoquinol PX (Zapata et al., 1998), in addition to scavenge H_2_O_2_, directly drains electrons from PET, contributing to alleviate the excess of reducing power that enhances the production of ROS but, at the same time, decreasing the efficiency of the use of light energy in photosynthesis. Similarly, the Mehler reaction generates $O_{2}^{∙-}$ by draining electron from PET (mainly from reduced non-heme iron-sulfur protein, FeSP_red_) and, thus, it decreases the efficiency of photosynthesis. At the photo-physical stage, the heath dissipation of excited chlorophyll to reduce the formation of ^1^O_2_ also impairs the use of light energy in photosynthesis. Therefore, plants have evolved mechanisms to regulate the processes of generation and scavenging of ROS by adjusting them to different environmental conditions in order to minimize ROS damage and maximize photosynthesis yield. The adjusting is got through appropriate level of zeaxanthin, SOD, and PX (Bowler et al., 1992; Casano et al., 1999; Eskling et al., 2001; Karpinski et al., 2001) for any combination of light intensity, temperature, and CO_2_ availability (which depends on several factors as stomatal opening).

However, the light intensity that receives the leaf strongly and rapidly fluctuates under natural conditions (Pearcy, 1994; Külheim et al., 2002), which make necessary continuous adjustments of enzyme activities and inevitable transitory burst of ROS and losses of photosynthesis efficiency. The adjustment is not easily reached because many processes are functionally interconnected in photosynthesis. Hence, like photophosphorylation, the dissipation of heat by zeaxanthin requires an appropriate gradient of proton (ΔpH) across the thylakoid membrane (Eskling et al., 2001). However, as pointed out by Heber and Walker (1992), when the components of PET are over-reduced the rate of cyclic PET is too low to supply the necessary extra transport of protons to thylakoid lumen; the functioning of cyclic PET requires balanced (poised) levels of both reduced and oxidized forms of the electron transporters. From this perspective, the draining of electrons from PET by Mehler reaction and plastoquinol PX allows to poise the redox level of the cyclic electron transporters (Casano et al., 2000) and, then, to maintain the appropriate ΔpH for the dissipation as heath of the excess of absorbed light. Thus, the generation of $O_{2}^{∙-}$ and H_2_O_2_ would be a less harmful alternative than the formation of ^1^O_2_ under transitory high light. The photo-inhibition of photosystem II (PSII; Osmond, 1994) is other important response of the photosynthetic machinery to transitory high light. When light comes back to moderate or low intensity, the recovery to full activity of photo-inhibited PSII could last several seconds and, frequently, minutes; too much time when compared with the rapid light intensity changes (fraction of second) that a section of a trembling leaf confronts frequently in windy fields. Quickly, after transition to low light, the ΔpH would collapse, now because the electron transporters of the cyclic PET become over-oxidized by the transitory low supply of electrons from PSII. The collapse is prevented because the redox poising of transporters is maintained through the feeding of electrons from NADH by the thylakoid Ndh complex (EC 1.6.5.3; Casano et al., 2000; Sabater and Martín, 2013) a product of the 11 plastid and a few nuclear *ndh* genes.

**FIGURE 2:**
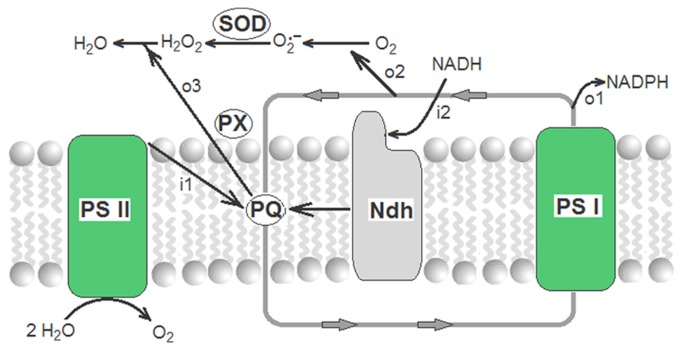
**Redox poising of electron transporters of the cyclic PET by chlororespiratory reactions.** Closed gray line with arrows corresponds to cyclic PET where only plastoquinone (PQ) and photosystem I (PSI) are shown. i1 and i2 are the influxes of electrons from, respectively, photosystem II (PSII) and from NADH (through the thylakoid Ndh complex). o1, o2, and o3 are the outfluxes of electrons to, respectively, NADP^+^, Mehler reaction, and plastoquinol peroxidase.

Therefore, operating alternatively, the supply of electrons (by the Ndh complex) and the drain of electrons (by concerted actions of the Mehler reaction, SOD and plastoquinol PX) ensure the fine-tuning of the redox level of the transporters of electrons in the cyclic PET. The complete sequence of reactions is:

$$2NADH+{2H}^{+}\,\;+\;2PQ\overset{\;\;Ndh\,\;complex\;\;\;}{\to }{2NAD}^{+}\,\;+\;2{PQH}_{2}$$

$$2{FeSP}_{oxid}+{PQH}_{2}\overset{\;\;\;\;\;\;\;\;\;\;\;\;\;PET\;\;\;\;\;\;\;\;\;\;\;\;}{\to }2{FeSP}_{red}+PQ+{2H}^{+}$$

$${2FeSP}_{red}+{2O}_{2}\overset{Mehler\,\;\;reaction}{\to }{2FeSP}_{oxid}+{2O}_{2}^{∙-}$$

$$H_{2}\,O_{2}\,\;+\;P\,Q\,H_{2}\overset{\;\;\;\;\;\;\;\;\;\;\;\;\;\;\;P\,X\;\;\;\;\;\;\;\;\;\;\;\;\;\;}{\to }2\,H_{2}\,O\,\;+\;P\,Q$$

$${2O}_{2}^{∙-}\,\;+\;{2H}^{+}\overset{\;\;\;\;\;\;\;\;\;\;\;\;\;SOD\;\;\;\;\;\;\;\;\;\;\;\;\;\;}{\to }O_{2}\,\;+\;H_{2}O_{2}$$

and results in a global respiratory process named chlororespiration (Casano et al., 2000; Joët et al., 2002; Nixon and Rich, 2007):

$$2NADH\,\;\;+\;{2H}^{+}\,\;+O_{2}\overset{\;\;\;\;\;\;\;\;\;\;\;\;\;\;\;\;\;\;\;\;\;\;\;\;\;\;\;\;\;\;\;\;}{\to }{2NAD}^{+}\,\;+\;{2H}_{2}O$$

that is schematized in **Figure 2**.

In the field, rapid increases and fluctuations of the intensity of light, high and low temperatures, hydric stress, and aggressive chemicals rapidly change the rates of electron supply (from PSII) and consumption (by the Benson–Calvin cycle). In this scenario, to maintain functional the cyclic PET and minimize damages by ROS, the activities of the chlororespiratory chain (Ndh complex, SOD, and PX) must be high in comparison with more stable or mild environments. Accordingly, the levels of the Ndh complex increase under different stresses (Serrot et al., 2008; Paredes and Quiles, 2013) and Ndh deficient tobaccos are especially sensible to damages under stress (Martín et al., 1996, 2004; Endo et al., 1999; Prochazkova et al., 2001; Rumeau et al., 2007). There is also a large and classic literature reporting the increase of SOD and/or PX under different stress conditions (see a revision in Bowler et al., 1992). In addition to other factors, the relative activities of the thylakoid Ndh complex, SOD, and PX must determine the relative levels the different ROS in the steady state. Therefore, when the activities were measured in parallel assays, levels of the thylakoid Ndh complex and of the chloroplast SODs (FeSOD and Cu/ZnSOD) and plastoquinol PX increase similarly in young photosynthesizing leaves of tobacco or barley plants subjected to diverse stressing agents (Casano et al., 1994, 1999; Martín et al., 1996, 2004). Always, increases of the Ndh, SOD, and PX are precisely mediated by ROS (Casano et al., 2001) at gene expression and, probably, enzyme activation (Lascano et al., 2003).

When adult-senescent leaves are subjected to different stresses, Ndh complex level and activity as well as those of plastid PX increase in a similar way than in young fully photosynthesis active leaves. In contrast, the induction of SOD in response to stress becomes progressively impaired when leaves enter senescence (Casano et al., 1994, 1999; Kurepa et al., 1997; Abarca et al., 2001a,b; Prochazkova et al., 2001; Ohe et al., 2005). The results provide clues for the frequently reported interactions between stress response and senescence (Behera et al., 2003) and suggest that the fail to induce chloroplastic SOD plays a key role in leaf senescence. This should strongly remember the animal systems where, transgenic over-expressing SOD and catalase show a significant life-span extension (Orr and Sohal, 1994). In the last, catalase must also be over-expressed to avoid the increase of H_2_O_2_ formed by over-expressed SOD. Chloroplast lacks catalase but contains several PXs (among them plastoquinol PX) whose increase in senescence must efficiently consume the low amount of H_2_O_2_ produced by chloroplast lacking SOD in senescing leaves. In this line, in adult-senescent leaves, over-expression of the Ndh complex and chloroplastic PX and under-expression of chloroplastic SOD must increase the level of $O_{2}^{∙-}$ and decrease the level of H_2_O_2_ in respect to the response of young leaves. As the Ndh complex provides electrons, *per se*, it must increase the redox level of the transporters and, consequently, the formation of ^1^O_2_ and $O_{2}^{∙-}$. In contrast to young leaves where increased formations of ^1^O_2_ and $O_{2}^{∙-}$ are neutralized, respectively, by increased heat dissipation and increased $O_{2}^{∙-}$ scavenging by higher SOD, in adult senescent leaves, ^1^O_2_ and $O_{2}^{∙-}$ produced after a mild stress increased continuously out of control because Ndh complex and PX are induced by ROS.

To the large number of classic publications reporting the decrease of chloroplastic SOD and the increase of PX during leaf senescence, many latter references report the increase of the Ndh complex during leaf senescence and fruit ripening (Martín et al., 1996; Casano et al., 1999, 2000; Lascano et al., 2003; Nashilevitz et al., 2010; Nilo et al., 2012; Serrot et al., 2012). In addition, the involvement of the Ndh complex in senescence seems clear from the delay of leaf senescence (some 30 days in respect to wild type) in transgenic tobacco defective in the *ndhF* gene and Ndh complex (Zapata et al., 2005). Significantly also, long-lived conifers, as *Pinus longaeva* whose needles remain functional for 35 years, lack *ndh* genes in their sequenced plastid DNA (Wu et al., 2011).

## HYPOTHESIS: A HIGH RATIO (^1^O_2_ + $O_{2}^{∙-}$)/H_2_O_2_ CHANGES STRESS DEFENSE RESPONSE TO PROGRAMMED LEAF SENESCENCE

Summarizing the evidences discussed above, the decrease of SOD and the increase of the Ndh complex play crucial roles in senescence by determining an increase of $O_{2}^{∙-}$, yet reported by McRae and Thompson (1983), and a decrease of H_2_O_2_ in chloroplasts which contrasts with the increase of these two ROS in stress responses. It seems that, within the network of transduction signals that regulate the response to stress, the nodes involved in the control of the levels of the Ndh complex remain intact in adult-senescent leaves but those controlling the levels of chloroplastic SOD (FeSOD and Cu/ZnSOD) fail to respond. Then, fine-tuning of the redox level of the electron transporters of cyclic PET is broken early in leaf senescence and gives way to their over-reduction and to a growing spiral of ROS production and death.

Externally supplied H_2_O_2_ increases the expression of the plastid *ndh* genes, the levels of the Ndh complex and the NADH dehydrogenase activity of the Ndh complex by phosphorylation of the NDH-F subunit (Casano et al., 2001; Lascano et al., 2003; Martín et al., 2009). In general, H_2_O_2_ is involved in the defense response against different biotic and abiotic stresses (Casano et al., 1999, 2001; Levine, 1999). Although H_2_O_2_ probably mediates the increase of the Ndh complex in the defense of young leaves against stress, the increase of the Ndh complex during senescence does not seem due to the increase of H_2_O_2_ production in chloroplasts. Bieker et al. (2012) reported that H_2_O_2_ level transitorily doubled in brassicaceae leaf during bolting and flowering time, probably related to decreases of catalase and ascorbate PX and not to a high production of H_2_O_2_ in chloroplasts. In fact, although H_2_O_2_ could increase in peroxisomes (Del Rio et al., 1998), there is no evidence of the increase of H_2_O_2_ production in chloroplasts during natural leaf senescence under field light and, accordingly, no accumulation of H_2_O_2_ was found during the PCD of bundle sheaths in the maize *camouflage1* (*cf1*) mutant (Huang and Braun, 2010). As pointed above, chloroplasts are probably the main cellular source of H_2_O_2_ in the leaf at light, and that source is considerably reduced in adult-senescent leaves. Reduced enough to abolish the main H_2_O_2_-dependent transduction signaling in the protection responses against stress. The hypothesis of a low level of H_2_O_2_ (in respect to ^1^O_2_ and $O_{2}^{∙-}$) during senescence is essentially based on the low levels of the activity that forms it (SOD) and the high level of the activity that consume it (PX). Rapid turnover and damage effects make difficult a precise definition of the level of ^1^O_2_ or $O_{2}^{∙-}$, of the (^1^O_2_ + $O_{2}^{∙-}$)/H_2_O_2_ ratio, and of a threshold ratio that changes defense response to PCD. At present, the concept of (^1^O_2_ + $O_{2}^{∙-}$)/H_2_O_2_ ratio urges researches on signaling downstream of ^1^O_2_ and $O_{2}^{∙-}$ and on methods for accurate measurements of ROS in plants. The determination of the levels of each ^1^O_2_, $O_{2}^{∙-}$, and H_2_O_2_ in cytosol and in each organelle is difficult but would be particularly relevant with data on the cellular location of the network nodes directly influenced by them.

In contrast to the other ROS produced in chloroplast (^1^O_2_, $O_{2}^{∙-}$, and HO^∙^), H_2_O_2_ is sufficiently stable and presumably permeable through chloroplast membranes to connect with the cytosolic network of signals that control specific gene expression in the nucleus. Immediate targets of H_2_O_2_ are not yet known and plausible candidates in cytosol, or also into the chloroplast, are 2-cysteine peroxiredoxins (PRDX). These could also act as redox signals (Dietz, 2003; Muthuramalingam et al., 2009; Puerto-Galán et al., 2013) and as regulators of the level of H_2_O_2_. Many evidences connect the H_2_O_2_ signaling with cascades of mitogen-activated protein (MAP) kinases that are involved in the H_2_O_2_ production and in the regulation of death, mainly in the response to biotic and abiotic stresses (Overmyer et al., 2003). Moreover, the H_2_O_2_-mediated increase of the *ndh* gene expression seems to depend on protein kinases (Casano et al., 2001; Lascano et al., 2003). However, no protein has yet identified that directly interacts with H_2_O_2_ in network signaling.

## SIGNALING DOWNSTREAM OF ^1^O_2_ AND $O_{2}^{∙-}$

If ^1^O_2_ and (or) $O_{2}^{∙-}$, in the place of H_2_O_2_ and its derivative HO^∙^, are the chloroplast ROS signals involved in programmed leaf senescence, the obvious question refers to its (their) immediate target. As mentioned above, ^1^O_2_ or $O_{2}^{∙-}$ can barely be exit from the chloroplast. Therefore, the immediate target must be into the chloroplast. The modification of amino acids is the main known damage produced by $O_{2}^{∙-}$ (**Figure 1**) and, essentially, the same amino acids are modified by $O_{2}^{∙-}$ and H_2_O_2_. Therefore, the oxidation of cysteine residues of proteins by $O_{2}^{∙-}$ could initiate in chloroplast a signal similar to those supposed for H_2_O_2_ in chloroplast and cytosol. However, no precise or specific protein target is yet known that initiates a signal after oxidation by $O_{2}^{∙-}$. Other possibility is that the general damage effects of $O_{2}^{∙-}$ in chloroplasts and the lack of H_2_O_2_ for draining the excess of electrons, should impair the rate of cyclic PET collapsing the thylakoid membrane potential and, thus, shooting the production of more ^1^O_2_ that would become the key initial signal that compels the cell to death at light. As pointed above, to maintain the leaf functional and healthy, the chloroplast forms other ROS than ^1^O_2_. Therefore, the low level of ^1^O_2_ must be an objective for enduring cell. For senescence and death, ^1^O_2_ probably initiates a signal chain linked to the damage of polyunsaturated fatty acids.

**FIGURE 3:**
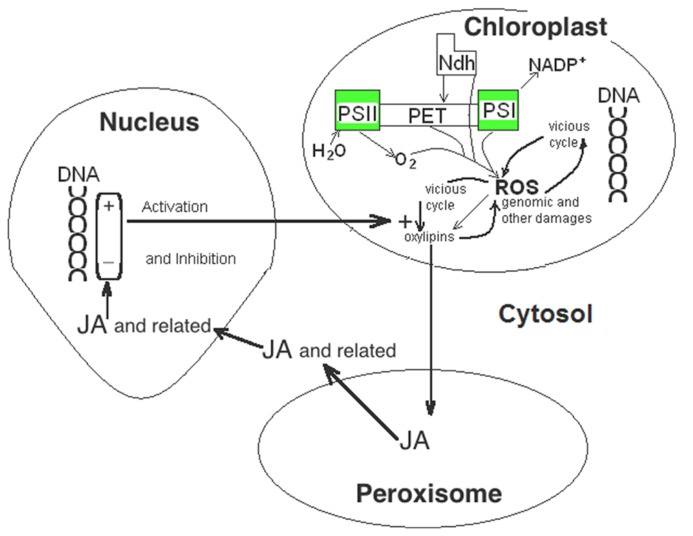
**Oxylipins facilitate the vicious cycle that increases the levels of ^1^O_2_ and $O_{2}^{∙-}$ (ROS).** JA and related compounds are formed in peroxisomes and cytosol from oxylipins generated in chloroplasts by the action of ^1^O_2_ on linoleic acid. Within nucleus, JA and related compounds inhibit genes for chloroplastic SOD and induce genes for chloroplastic LOX and for the Ndh complex subunits encoded in the nucleus that further increase the levels of ^1^O_2_ and $O_{2}^{∙-}$ ROS and, then, of JA.

As **Figure 1** shows, ^1^O_2_ attacks polyunsaturated fatty acids. The first products of the attack of polyunsaturated fatty acids of membrane lipids by ^1^O_2_ are 13-hydroperoxy derivate fatty acids. Among them, 13-hydroperoxy linoleic acid is transformed in chloroplasts to the oxylipin (9S, 13S)-12-oxo-phytodienoic acid, which is transformed into jasmonic acid (JA) in peroxisomes and further, in cytosol, to related compounds as methyl jasmonate (Creelman and Mulpuri, 2002; Wasternack, 2007). In this way, JA and several related compounds mediate the rapid response to the stress generated by the production of ^1^O_2_ (Wagner et al., 2004) inhibiting the synthesis of protein for the photosynthetic machinery (Reinbothe et al., 1993) and stimulating the expression of genes for the defense against stress and of the senescence associated genes (SAG; Creelman and Mulpuri, 2002). Among other effects, JA and related oxylipin derivatives stimulate the expression of chloroplast lipoxygenase (LOX; Bachmann et al., 2002) and increase the level of the thylakoid Ndh complex (Cuello et al., 1995). Precisely, LOX catalyzes the reaction of free linoleic acid with O_2_ to form further 13-hydroperoxy linoleic, which, as described, generates more JA and derivatives (Schaller et al., 2004), now dependent on O_2_, but not of the ^1^O_2_ formed by light excess. Therefore, under appropriate conditions, which are plausibly related to the levels of other signals, JA seems a key signal able to generate an autocatalytic cell path to death (**Figure 3**). In this regard, it is significant that treatments with JA increase the production of ^1^O_2_ (Guo et al., 2010) and accelerates senescence (Wasternack, 2007). If not the start gunfire of senescence, the coincidence of high PX, Ndh complex, LOX, and ^1^O_2_ with low SOD seems to open the irreversible path to cell death. The spiral increase of ROS (and more precisely of ^1^O_2_ or $O_{2}^{∙-}$) is under genetic control, firstly determining the expression of PX and Ndh complex, and later of LOX. The last initiates a light independent increase of JA and, as a consequence, of further light-dependent increase of ^1^O_2_. Key nodes of the death path would be JA, which increase and thus induces SAG, and H_2_O_2_, which decreases and thus prevent the induction of stress defense genes. Independently of the damage effect of ROS, through the further induction of SAG by JA, the overall process appears a PCD.

It would be concluded that the initiation of the program for leaf death is the fail to properly stimulate the expression chloroplastic SODs under mild stress, but the meaning of starting point is fading in an autocatalytic spiral of events as that proposed. Hence, among SAGs, MYB transcription factors have been described (Chen et al., 2002; Buchanan-Wollaston et al., 2005) that bind to the AACTAA motif inhibiting the progression of the transcription machinery to downstream genes. Precisely, the AACTAA motif is close to AGATAA and TATA motifs for the initiation of transcription in upstream region of the *Csd*2 gene (encoding the chloroplast Cu/ZnSOD) in *Arabidopsis*. In this way, the induction of the SAG for MYB must obstruct the expression of the chloroplastic SOD, hence contributing, in addition to the mechanisms described above, to the growing spiral of the ^1^O_2_ and $O_{2}^{∙-}$ ROS in leaf senescence (Sabater and Martín, 2013).

The regulatory upstream region of the *Arabidopsis* *Csd2* gene also includes two W-boxes (TGAC(T)) recognized by WRKY transcription factors (Eulgem et al., 2000), some of them induced during leaf senescence (Singh et al., 2002). The gene encoding one of them, *WRKY53*, also has a high relevance as the crossing point where different signals, and more specifically JA and H_2_O_2_ (Zentgraf et al., 2010) should determine defense response or PCD. In effect, although other signals as salicylic acid and nitric oxide (Wang et al., 2013) may be involved, the model described above indicates that the high ^1^O_2_ plus $O_{2}^{∙-}$ /H_2_O_2_ ratio in senescence, in respect to non-lethal stress response, is transduced into a high comparative JA/H_2_O_2_ ratio. JA and H_2_O_2_ would influence (by activation or inhibition, depending on concentration and other factors) at the different steps of complex transduction networks that control stress response and PCD by WRKY53. To add more complexity, several proteins control the expression of the *WRKY53* gene and the binding of the WRKY53 protein to DNA upstream of genes expressed in senescence. Therefore, through variable responses to JA and H_2_O_2_, the complex network of WRKY proteins could be crucial determining the transition of the defense response to PCD as a consequence of increased ^1^O_2_ plus $O_{2}^{∙-}$ /H_2_O_2_ ratio.

The model described for leaf senescence requires, at least in its first stages, light acting on the photosynthesis machinery. But the syndrome of leaf senescence would be more complex in field where alternating light and dark periods during the day could overlap light dependent senescence with dark dependent senescence. Under this condition, the involvement of a high production of H_2_O_2_ in cytosol or non-chloroplast organelles could be relevant. In addition, hormonal factors related to developmental processes, as seed filling and shadowing by upper leaves, and nutritional factors can produce a mixture senescence mechanisms, one of which could be dominant in some plants and environments.

## CONCLUDING REMARKS AND FUTURE PROSPECTS

In contrast to animals, the development of plants is strongly affected by environmental factors and, not surprisingly, ROS are involved as signals in developmental processes leading to cell death and in the defense response against environmental stress. Difficulties to determine the levels of some specific ROS in the different cell compartments open key questions as the levels and mechanisms through which ROS control different issues such as death and defense. In addition, and similarly to other signals of the networks controlling developmental processes, the level of ROS are subjected to multiple cross, feed-forward, and feed-back effects and poorly known factors, as iRNA and epigenetic modifications, that make difficult to identify a precise cause to effect chain of events explaining the final response of the plant.

However, in contrast to most other signals, the successive steps for enzymatic, and non-enzymatic generation and scavenging of the main ROS (^1^O_2_, $O_{2}^{∙-}$ , H_2_O_2_, and HO^∙^ ) are well known. In addition, the levels of the enzymes involved in the generation and scavenging of most ROS can be accurately determined in different cell compartments and in different stress and senescence conditions. From enzyme data, qualitative level differences between stress and senescence for each ROS can reasonably be proposed for different cell compartments which progresses in ROS determination (Woolley et al., 2013) could test in the future. With this approach, the hypothesis presented here of a high ratio (^1^O_2_ + $O_{2}^{∙-}$)/H_2_O_2_ for the initiation of senescence at light, could be extended or modified after comparison of enzymatic activities in stress responses with those in other types of senescence, including the senescence of non-photosynthetic tissues. The results must facilitate the identification of proteins that directly interact with specific ROS in the regulatory cellular networks. In a first approach, the complex of WRKY proteins deserves full attention. In addition, the results would provide a wide perspective to investigate more precisely the control of genes for generation and scavenging of ROS in stress and PCD.

## Conflict of Interest Statement

The authors declare that the research was conducted in the absence of any commercial or financial relationships that could be construed as a potential conflict of interest.
